# The combination of aldehyde dehydrogenase 1 (ALDH1) and CD44 is associated with poor outcomes in endometrial cancer

**DOI:** 10.1371/journal.pone.0206685

**Published:** 2018-10-29

**Authors:** Hsin-Hui Huang, Yu-Chi Wang, Yu-Ching Chou, Mu-Hsien Yu, Tai-Kuang Chao

**Affiliations:** 1 Department of Obstetrics and Gynecology, Tri-Service General Hospital, National Defense Medical Center, Taipei, Taiwan; 2 School of Public Health, National Defense Medical Center, Taipei, Taiwan; 3 Department of Pathology, Tri-Service General Hospital, National Defense Medical Center, Taipei, Taiwan; University of South Alabama Mitchell Cancer Institute, UNITED STATES

## Abstract

Aldehyde dehydrogenase 1 (ALDH1) and CD44 have been established as biomarkers for predicting the survival of many types of cancer patients. This study evaluated the expression and clinical significance of these putative cancer-cell markers in a series of tumor samples from endometrial cancer (EC) patients using tissue microarray. We examined 245 endometrial samples, including 132 (53.87%) pre-malignancy lesions and 113 (46.12%) malignant endometrial lesions from biopsies or hysterectomies. We examined the expression of CD44 and ALDH1 in these samples using immunohistochemistry staining. Correlations in the relative expression of these markers with clinicopathological parameters were also assessed. A high level of expression of ALDH1 was found in 44.25% (50/113) of the endometrial cancer samples, which was significantly correlated with a poor overall survival rate (p = 0.035). High-level CD44 expression was found in 35.4% (40/113) of the cases and was also correlated with a poor overall survival rate (p = 0.035). A simultaneous high expression of both markers was correlated with an extremely poor overall survival (p = 0.013). Our results show that tumors with higher expressions of both ALDH1 and CD44 were related to a poorer overall survival rate among EC patients. The combination of ALDH1 and CD44 could be a promising marker for developing additional targeted therapy for severe endometrial cancers.

## Introduction

Endometrial cancer (EC) is one of the most common gynecological malignancies globally, with an estimated incidence of 60,050 patients and 10,470 deaths in the USA in 2016 alone [1, 2]. EC is usually diagnosed at an early stage, with approximately 80% of cases being diagnosed in stage I. The National Cancer Intelligence Network has stated that 83–84% of EC are diagnosed early, with 74–75% of these in stage I.

The most common symptom of EC is abnormal uterine bleeding, which presents in 90% of women, and is the main reason that this cancer is usually discovered in early stages. Although the 5-year overall survival (OS) of EC is as high as 88% [3], there are some patient subgroups who have lower survival rates, higher recurrence rates, and poorer clinical outcomes. Therefore, it is important to discover new and more specific biomarkers to identify these patients and to facilitate accurate diagnoses, enhance prognostic predictions, and contribute to individualized treatments to improve patient survival rates.

Currently, the role of biomarkers in EC has not been clearly defined. CA125 is one of the most commonly used biomarkers, but it lacks sensitivity and specificity for detection and predicting prognoses [4–6]. Aldehyde dehydrogenase 1 (ALDH1) is a predominant isoform of the *ALDH* family in mammals, and it has recently been reported as a novel marker in gynecologic cancer. Rao et al. found it to be useful in cervical cancer [7], and Huang et al. found an association with prognosis in ovarian cancer [8]. In addition, overexpression of ALDH1 suggests poor prognosis of patients with endometrioid adenocarcinoma, which may imply that ALDH1 indicates the presence cancer-initiating cells [9]. Our previous study demonstrated that high ALDH1 expression in ovarian cancer patients correlates with a poor prognosis [10].

CD44 is an adhesive molecule and a cell-surface glycoprotein. It is highly expressed in many malignancies, and its recruitment to the cell surface can regulate cancer metastasis [11]. The role of CD44 has been examined in cancers such as leukemia, colon cancer, and breast cancer. Cells with high CD44 expression are considered to have tumorigenic behavior, and their presence can be seen as an early marker for neoplastic stem cell proliferation [12]. However, the role of combined ALDH1 and CD44 expression in EC is unclear, and the prognostic value of these two markers also requires additional research to reach more conclusive results. This study investigated the expression of both ALDH1 and CD44 in EC and their prognostic value for EC patients. We enrolled 113 patients with EC and evaluated the prevalence of ALDH1 and CD44 expression in premalignant and malignant endometrial lesions, as well as the associations of both markers with clinicopathological parameters and OS.

## Materials and methods

### Patient and specimens

A total of 245 samples comprising 132 pre-cancerous lesions and 113 EC tumors were examined. Among patients representing the collected EC samples, 21 were lost to follow-up and no survival data could be obtained. Paraffin-embedded tissues were retrieved from the Department of Pathology of the Tri-Service General Hospital, and tissue microarray slides were prepared according to a published method [13]. This study was approved by the Institutional Review Board of the Tri-Service General Hospital (TSGHIRB No: 100-05-042 and 2-101-05-041) and obtained written consent to approve this consent procedure. Informed consent was obtained from all patients.

### Tissue microarray (TMA)

One tissue core (2 mm in diameter) was taken from each of the representative tissue samples and placed in a new recipient paraffin block. All tumors were pathologically staged according to the 2013 TNM system.

### Immunohistochemistry (IHC)

Tissue microarray sections were de-waxed in xylene, rehydrated in alcohol, and immersed in 3% hydrogen peroxide for 10 min to suppress endogenous peroxidase activity. Antigen retrieval was performed by heating each section at 100°C for 30 min in 0.01 mol/L of sodium citrate buffer (pH 6.0). After rinsing three times for 5 min each in phosphate buffered saline (PBS), the sections were incubated for 1 h at room temperature with mouse ALDH1 (1:100; clone 44/ALDH; BD Biosciences, Franklin Lakes, NJ, USA) and CD44 (1:100; ab51037; Abcam Biotech, Cambridge, UK) diluted in PBS.

After washing three times for 5 min each in PBS, the sections were incubated with horseradish peroxidase-labeled immunoglobulin (Dako, Carpinteria, CA, USA) for 1 h at room temperature. After washing three additional times, the peroxidase activity was visualized with a solution of diaminobenzidine (DAB) at room temperature. The immunoreactivity and histological appearance of all tissue microarray slides were examined and scored independently and concurrently by two authors (both gynecological pathologists). The immunoreactivity was graded arbitrarily and semi-quantitatively by considering the intensity and percentage of staining on the tissue microarray slides, as described previously.

The ALDH1 and CD44 intensity of individual tumor cells was scored as 0 (no staining), 1+ (weak intensity), 2+ (moderate intensity), or 3+ (strongest intensity). The percentages of cells with ALDH1 and CD44 staining at each intensity were also estimated (range, 0–100). For the semiquantitative analysis of the ALDH1 and CD44 production, the absolute ALDH1 and CD44 scores were calculated by multiplying the estimated percentages of stained cells at each intensity by the corresponding intensity value, which produced immunostaining scores ranging from 0–300.

To compare the absolute ALDH1 and CD44 scores between different endometrial lesions, the optimal cut-off values of the ALDH1 and CD44 f-scores were determined using receiver operating characteristic curve analysis. ALDH1^Low^ was defined as a score below 10, and ALDH1^High^ was defined as a score higher than 10. CD44^-^ was defined as a score of 0, and CD44^+^ was defined as a score higher than 0. As a negative control, slides were treated by replacing the primary antibody with non-immune serum.

### Statistical analysis

All values are expressed as mean ± standard error of the mean (SEM) and percentages. Analysis of variance (ANOVA) and the chi-square test were used to compare the expression of ALDH1 and CD44 between groups with a normal endometrium, endometrial hyperplasia (EH) without atypia, hyperplasia (AH), and EC. The chi-square test or Fisher’s exact test was used to identify trends and differences in distribution between ALDH1 and CD44 expression and clinicopathological characteristics.

The OS time was assessed by Cox regression analysis. Kaplan-Meier survival curves were compared using the log-rank test. A two-sided *p*<0.05 was considered significant. All analyses were performed using SPSS statistics software for Windows (version 21; IBM Corp, Armonk, NY).

## Results

The 245 samples collected in this study included 42 normal endometrium samples, 48 EH samples, 42 EH samples with atypia or AH, and 113 EC samples. Among the 113 EC samples, 82 were endometrioid adenocarcinoma (EmAC), 19 were serous carcinoma (SC), 10 were clear cell carcinoma (CC), and 2 were mucinous carcinoma (MC). Follow-up data were available for 92 patients, while 21 patients were lost to follow-up.

A total of 113 patients with suspected EC had tumor samples analyzed for the prevalence of ALDH1 and CD44 expression, as well as the associations of both markers with patient clinicopathological parameters and OS. We used IHC staining and analyzed expression based on the staining area and the intensity of color reaction for these two putative biomarkers. ALDH1 was mainly expressed in the cytoplasm, whereas the CD44 staining was mainly on the cell membranes of tumor cells, as expected based on the usual distribution. ALDH1 expression was assessed in tumor cells and stromal cells. By evaluating the prevalence of ALDH1 and CD44 expression in normal, pre-malignant, and malignant endometrial lesions, we observed that both markers (44.45% and 35.40% of EC patients expressed ALDH1^High^ and CD44^+^, respectively, p<0.001) showed higher expression in EC samples than in either normal or pre-malignant lesions (Table 1). However, there were no differences in the distribution of ALDH1 (p = 0.52) and CD44 (p = 0.91) expression between the EH and AH groups.

**Table 1:** Chi-square test results for ALDH1 and CD44 expression score based on the staining area and intensity of color reaction.

	Normal no. (%)	EH no. (%)	AH no. (%)	EC no. (%)	*p* ^*a*^ value
ALDH1					<0.001
Low	40 (95.24)	40 (83.33)	37 (88.10)	63 (55.75)	
High	2 (4.76)	8 (16.67)	5 (11.90)	50 (44.25)	
CD44					<0.001
- (negative)	40 (95.24)	47 (97.92)	40 (95.24)	73 (64.60)	
+ (positive)	2 (4.76)	1 (2.08)	2 (4.76)	40 (35.40)	
Combination of ALDH1 & CD44					<0.001
ALDH1^Low^ & CD44^−^	38 (90.48)	39 (81.25)	36 (85.71)	44 (38.94)	
(ALDH1^Low^ & CD44^+^)+(ALDH^High^ & CD44^−^)	4 (9.52)	9 (18.75)	5 (11.90)	48 (42.48)	
ALDH1^High^ & CD44^+^	0 (0)	0 (0)	1 (2.38)	21 (18.58)	

Normal: normal endometrium; EH: endometrial hyperplasia without atypia; AH: atypical hyperplasia; EC: endometrial carcinoma.

^a^ Fisher’s exact test.

**Fig 1:**
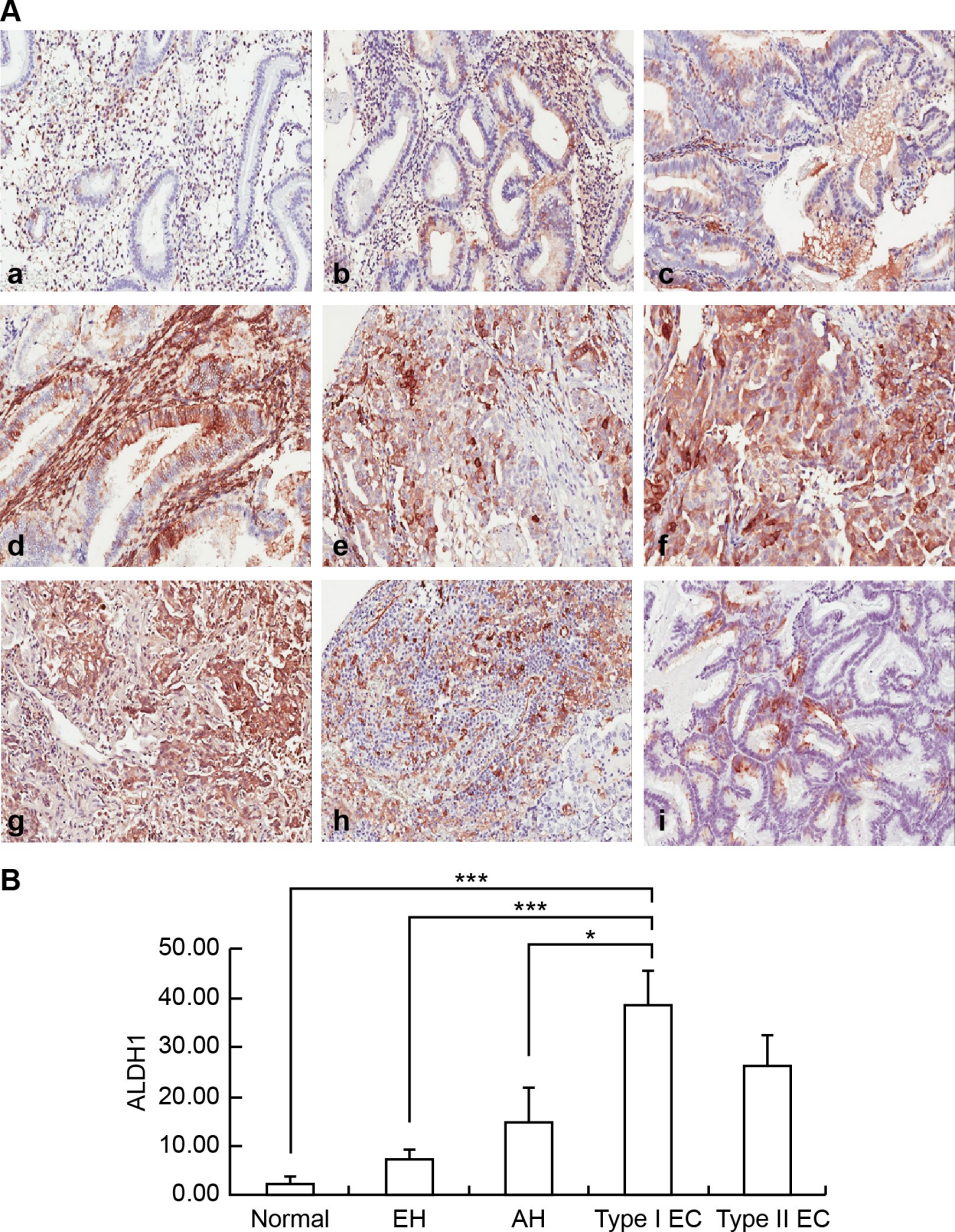
Examples of immunohistochemical staining for ALDH1 in endometrial lesions. (A) Immunohistochemical examination of ALDH1 expression in normal endometrium (a), EH without atypia (b), AH (c), grade 1 EmAC (d), grade 2 EmAC (e), grade 3 EmAC (f), SC (g), CC (h), and MC (i). (B) Semiquantitative Comparison of ALDH1 Immunostaining Scores Between Normal Endometrium, EH without Atypia, AH, and Type I, and Type II EC. Normal endometrium vs. Type I EC, p<0.001***; EH without atypia vs. Type I EC, p<0.001***; AH vs. Type I EC, p<0.05*.

ALDH1 protein expression was significantly higher in type I EC than in normal endometrium and pre-malignant endometrial lesions (Fig 1A). A semiquantitative analysis of ALDH1 immunostaining was also performed, and there was a significant difference in ALDH1 scores between the groups with pre-malignant and malignant endometrial lesions (p<0.001; Fig 1B). However, the difference between type I and type II EC (Fig 1A) was not significant (Fig 1B). In contrast, strong stromal expression of ALDH1 had a statistically significant association with pre-malignant endometrial lesions compared to EC (p<0.001) (S1 Table).

**Fig 2:**
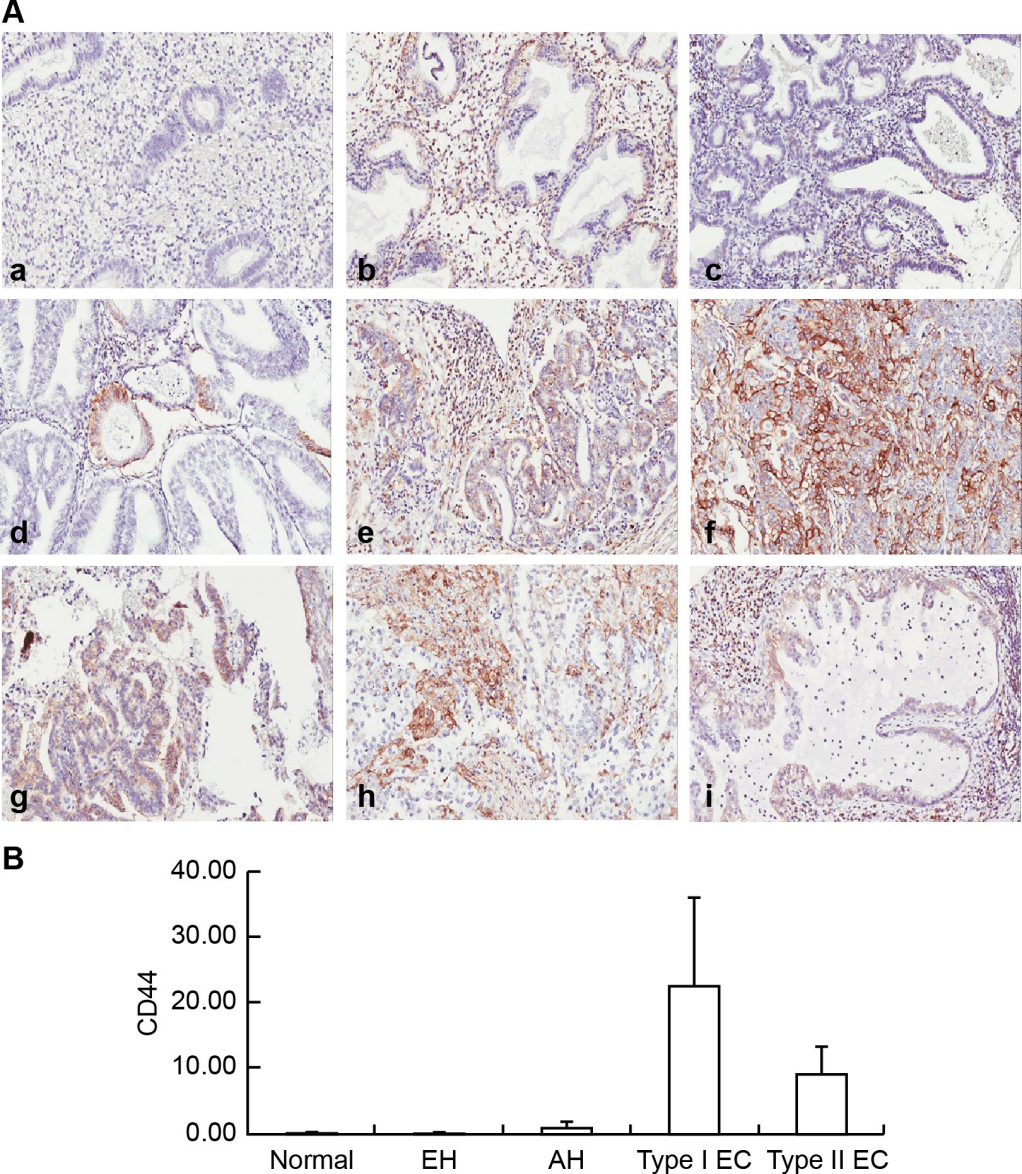
Examples of immunohistochemical staining for CD44 in endometrial lesions. (A) Immunohistochemical examination of CD44 expression in normal endometrium (a), EH without atypia (b), AH(c), grade 1 EmAC (d), grade 2 EmAC (e), G3 EmAC (f), SC (g), CC (h), and MC (i). (B) Semiquantitative Comparison of CD44 Immunostaining Scores Between Normal Endometrium, EH without Atypia, AH, and Type I, and Type II EC. One-way ANOVA, p = 0.229.

In the scoring of CD44 staining in the endometrium as shown in Fig 2A and Table 1, CD44^+^ was observed in only 2/42 samples of normal endometrium (4.76%), 1/48 samples of EH without atypia (2.08%), and 2/42 AH samples (4.76%). However, CD44^+^ was observed in 40/113 EC samples (35.4%). A semiquantitative analysis was also performed for CD44^+^ immunostaining. Despite the high presentation of CD44^+^ in EC cells, there was no significant difference in the CD44^+^ scores between the groups with pre-malignant and malignant endometrial lesions (p = 0.229; Fig 2B).

As shown in Table 2, there were no significant associations found between ALDH1^High^ and ALDH1^Low^ with tumor FIGO stage (p = 1.000), nuclear grade (p = 0.651), or EC subtype (p = 0.781) for the various histological type comparisons. There was also no significant difference in the distribution of ALDH1^High^ and ALDH1^Low^ (p = 0.557) when comparing type I EC (EmAC G1 and G2) plus mucinous carcinoma with type II EC (CC, SC) plus EmAC G3. Furthermore, the expression of CD44 was significantly associated with nuclear grade (p = 0.003) and subtype of EC (p = 0.012 for the various histological type comparisons, and p = 0.019 when comparing type I EC [EmAC G1 and G2] plus mucinous carcinoma with type II EC (CC, SC) plus EmAC G3). There was also a marginally significant association between patient age and CD44 expression (p = 0.049). Table 3 show the results of the combination of ALDH1 and CD44 expression analysis in EC patients. There were no significant associations between ALDH1 and CD44 expression with age, FIGO stage, or EC subtype, and there was only a marginally significant association with nuclear grade (p = 0.048). It appears that those with both ALDH1 and CD44 expression more than median had a higher proportion of type II EC (CC, SC) plus EmAc G3 on histology. While statistically not significant, it is only marginally insignificant and is worth mentioning.

**Table 2:** Correlation of ALDH1 and CD44 expression score with clinicopathological features in endometrial carcinoma.

	ALDH1^Low^	ALDH1^High^		CD44^-^	CD44^+^	
Characteristic	≤10	>10	*P* value	-	+	*P* value
**Patients (no.)**	52	40		58	34	
**Age (years)**			0.370			0.049
**Range**	31–80	33–88		31–83	33–88	
**Mean±SEM**	54.35±1.40	56.65±2.13		53.52±1.40	58.47±2.20	
**FIGO Stage [no. (%)]**			1.000			0.537
**I, II**	36 (57.10)	27 (42.90)		41 (65.1)	22 (34.9)	
**III**	15 (55.60)	12 (44.40)		15 (55.6)	12 (44.4)	
**Nuclear grade [no. (%)]**			0.651			0.003
**Grade 1**	24 (61.54)	15 (38.46)		32 (82.05)	7 (17.95)	
**Grade 2**	15 (55.56)	12 (44.44)		15 (55.56)	12 (44.44)	
**Grade 3**	13 (50.00)	13 (50.00)		11 (42.31)	15 (57.69)	
**Histological type [no. (%)]**			0.781^a^			0.012^a^
**Clear cell carcinoma**	3 (50.00)	3 (50.00)		1 (16.67)	5 (83.33)	
**Endometrioid Adenocarcinoma**	41 (56.94)	31 (43.06)		50 (69.44)	22 (30.56)	
**Mucinous Carcinoma**	2 (100.00)	0 (0)		2 (100.00)	0 (0)	
**Serous Carcinoma**	6 (50.00)	6 (50.00)		5 (41.67)	7 (58.33)	
**Histological type [no. (%)]**			0.557			0.019
**Type I EC (EmAC G1 and G2) + MC**	39 (59.1)	27 (40.9)		47 (71.2)	19 (28.8)	
**Type II EC (CC, SC) + EmAC G3**	13 (50.0)	13 (50.0)		11 (42.3)	15 (57.7)	

^a^ Fisher’s exact test.

CC: clear cell carcinoma; EmAC: endometrioid adenocarcinoma; MC: mucinous carcinoma; SC: serous carcinoma.

**Table 3:** Correlation of combination of ALDH1 and CD44 expression score with clinicopathological features in endometrial carcinoma.

	Both	At least one	Both	
Characteristic	≤ median	> median	> median	*P* value
Patients (no.)	36	38	18	
Age range (years)	31–77	40–83	33–88	0.162
Mean ± SEM	52.72±1.67	56.16±1.77	58.89±3.63	
FIGO Stage [no. (%)]				0.777
I, II	26 (41.3)	25 (39.7)	12 (19.0)	
III	9 (33.3)	12 (44.4)	6 (22.3)	
Nuclear grade [no. (%)]				0.048
Grade 1	21 (53.85)	14 (35.90)	4 (10.26)	
Grade 2	8 (29.63)	14 (51.85)	5 (18.52)	
Grade 3	7 (26.92)	10 (38.46)	9 (34.62)	
Histological type [no. (%)]				0.165^a^
Clear cell carcinoma	1 (16.67)	2 (33.33)	3 (50.00)	
Endometrioid Adenocarcinom	30 (41.67)	31 (43.06)	11 (15.28)	
Mucinous Carcinoma	2 (100.00)	0 (0)	0 (0)	
Serous Carcinoma	3 (25.00)	5 (41.67)	4 (33.33)	
Histological type [no. (%)]				0.059
Type I EC (EmAC G1 and G2) + MC	29 (43.9)	28 (42.5)	9 (13.6)	
Type II EC (CC, SC) + EmAC G3	7 (26.9)	10 (38.5)	9 (34.6)	

^a^ Fisher’s exact test.

SEM: standard error of the mean, CC: clear cell carcinoma; EmAC: endometrioid adenocarcinoma; MC: mucinous carcinoma; SC: serous carcinoma.

**Fig 3:**
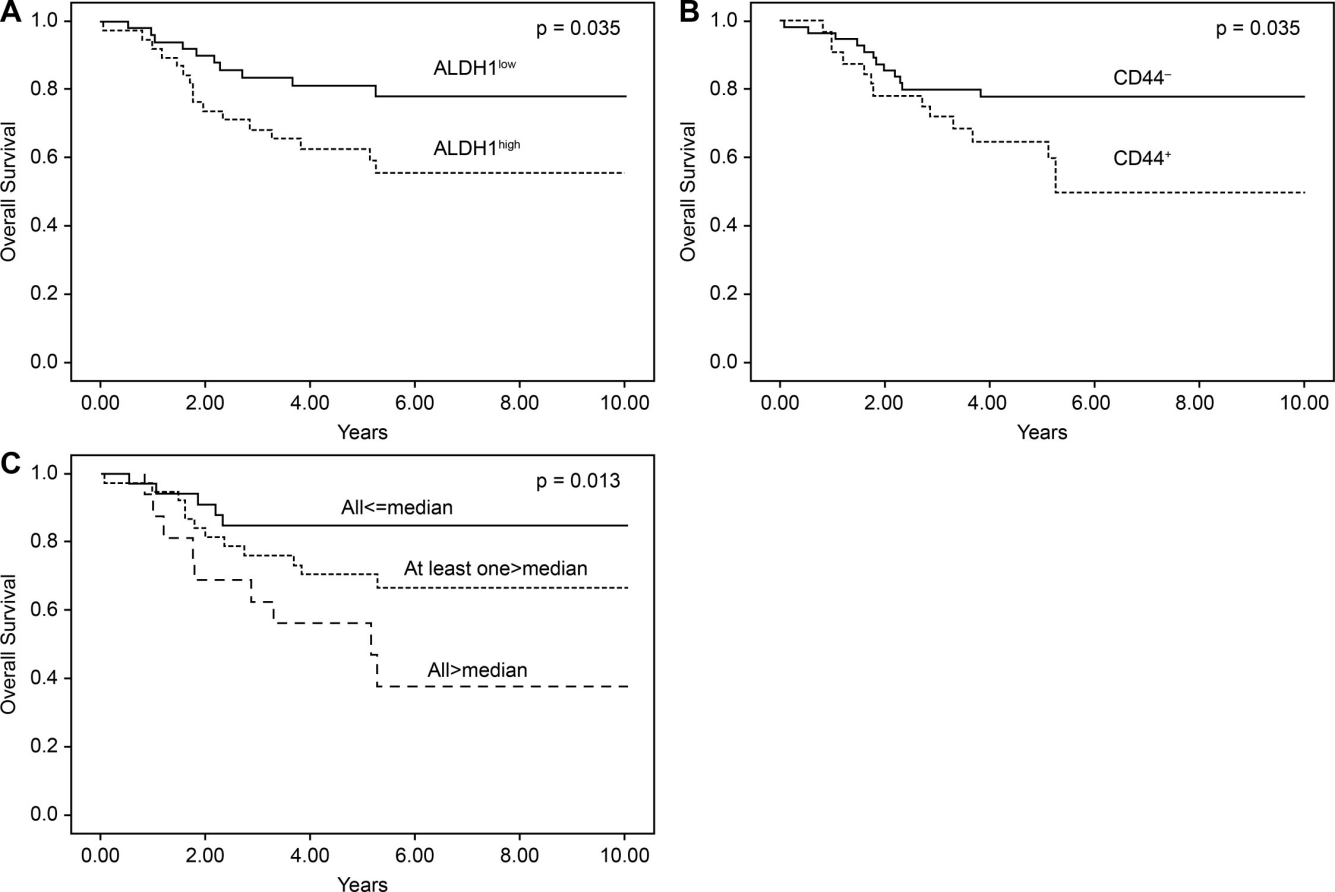
Kaplan–Meier analysis of survival in patients with EC based on ALDH1 and CD44 protein immunostaining. Patients with a higher ALDH1 (A), CD44 (B), combine ALDH1 and CD44 (C) immunostaining score had poor overall survival compared with those with a lower immunostaining score in patients with EC.

The results of the Kaplan-Meier survival analysis were stratified according to ALDH1 and CD44 score, as shown in Fig 3. Patients with ALDH1^High^ had poor OS rates compared to those with ALDH1^Low^ in the entire cohort (p = 0.035), as well as in patients with CD44^+^ (p = 0.035). In the combination analysis of ALDH1^High^ and CD44^+^, the higher presentation of both markers was correlated with poor OS (p = 0.013).

The multivariate analysis revealed that higher ALDH1 levels conferred a hazard ratio of 2.28 (95% confidence interval (CI), 1.04–5.03), while higher CD44 levels conferred a hazard ratio of 2.25 (95% CI, 1.04–4.87). Higher combined ALDH1^High^ and CD44^+^ expression levels conferred a hazard ratio of 4.61 (95% CI, 1.54–13.78), and a higher nuclear grade conferred a hazard ratio of 3.21 (95% CI, 1.33–7.78). Type II EC conferred a hazard ratio of 3.31 (95% CI, 1.53–7.16). There was a significant correlation between pathologic stage and OS. The Cox proportional hazards regression analysis was adjusted for age, stage, and histological grade. The results revealed an independent effect of ALDH1^High^ and CD44^+^ on OS, with higher combined ALDH1 and CD44 levels having higher hazard ratio (HR: 3.42, 95% CI, 0.97–12.05) (Table 4).

**Table 4:** Multivariate survival analysis of clinicopathological factors in 92 EC patients.

Variable	Univariate analysis crude HR (95% CI)	*P* value	Multivariate adjusted HR (95% CI)	*P* value
Age (years)	1.04 (1.01–1.07)*	0.024	1.03 (0.99–1.06)	0.130
ALDH1 expression^a^				
Low	1.00 (Ref.)			
High	2.28 (1.04–5.03)*	0.041	—	
CD44 expression^b^				
− (negative)	1.00 (Ref.)			
+ (positive)	2.25 (1.04–4.87)*	0.040	—	
ALDH1 and CD44 expression				
Both ≤median	1.00 (Ref.)		1.00 (Ref.)	
At least one >median	2.23 (0.79–6.33)	0.133	2.14 (0.66–6.78)	0.204
Both >median	4.61 (1.54–13.78)**	0.006	3.42 (0.97–12.05)#	0.056
FIGO Stage				
I, II	1.00 (Ref.)		1.00 (Ref.)	
III, IV	2.56 (1.14–5.75)*	0.022	1.50 (0.60–3.76)	0.382
Nuclear grade				
Grade 1	1.00 (Ref.)			
Grade 2	0.93 (0.30–2.84)	0.895	—	
Grade 3	3.21 (1.33–7.78)*	0.010	—	
Histological type				
Type I EC (EmAC G1 and G2) + MC	1.00 (Ref.)		1.00 (Ref.)	
Type II EC (CC, SC) + EmAC G3	3.31 (1.53–7.16)**	0.002	2.76 (1.09–6.86)*	0.032

^a^Low expression of ALDH1 is defined as ≤10; high expression of ALDH1 is defined as >10.

^b^CD44-negative is defined as score = 0; CD44-positive is defined as score >0.

CI: confidence interval; EC: endometrial cancer; HR: hazard ratio; Ref: reference group; EmAC: endometrioid adenocarcinoma; MC: mucinous carcinoma; CC: clear cell carcinoma; SC: serous carcinoma.

^#^p<0.1

*p<0.05

**p<0.001.

## Discussion

This study evaluated the expression and clinical significance of ALDH1 and CD44 expression levels in a series of samples from patients with suspected EC. To the best of our knowledge, this is the first study to examine the IHC expression of these two putative cancer stem cell markers, ALDH1 and CD44. The study was performed using a TMA method on samples from 113 EC patients. We evaluated the correlation between clinicopathological parameters and the expressions of each marker. Our findings demonstrated that higher expression of ALDH 1 and CD44 in EC cells is associated with a poorer OS rate.

The current treatment for EC is surgery, either with or without subsequent adjuvant radiotherapy or chemotherapy. Adjuvant therapy may improve progression-free survival in patients with advanced or recurrent cancer. Although the 5-year OS rate can be as high as 88% [3], there are some patients who have poorer response to treatment, higher rates of recurrence, and poor OS.

ALDHs are a family of intracellular enzymes that are involved in cellular detoxification, differentiation, and drug resistance by oxidation of cellular aldehydes [14]. Cells with clonogenic, self-renewing, differentiating, and tumorigenic properties have been found in endometrial carcinoma tumor cells, which suggests that cancer stem cells play an important role in EC. The major function of ALDHs is aldehyde detoxification, which serves to protect stem cells against the destructive properties of oxidative aldehydes. It has been shown that both human and murine hematopoietic and neural stem cells, as well as related progenitor cells, exhibit high ALDH activity [15–18]. ALDH1 has been shown to be a marker of cancer stem cells in many solid tumors, including head and neck [19], pancreas, lung [20], liver [21], ovary [22], and colon carcinoma [23]. In a review article by Januchowski et al. [24], an association was revealed between the strong expression of ALDH1 and poor prognosis in patients with breast [25, 26], prostate [27], and bladder cancer [28]. ALDH1 has also been considered to be related to poor prognosis in endometrioid adenocarcinoma and is a candidate marker of cancer-initiating cells [9]. Another study using a tissue array of serous ovarian cancer showed that high ALDH1 expression was correlated with shorter disease-free and OS, compared to those with low ALDH1 expression [22]. ALDH1 has also been considered an independent prognostic factor for disease-free survival and OS in patients with brain cancer [29]. Moreover, there was an inverse statistically significant association with strong stromal positivity of ALDH1 in pre-malignant endometrial lesion compared to EC (p<0.001). ALDH1 staining in stromal cells might result from the presentation of ALDH1 protein by dendritic cells known to attenuate tumor outgrowth [30]. Additionally, it might be reasoned that ALDH1 expressed in different types of cells is involved in different molecular pathways.

CD44, a transmembrane protein, has been shown to be a reliable stem cell marker based on its ability to isolate a sub-population of cells displaying stem cell properties from normal human endometrial tissue and endometrial carcinomas. However, the expression levels of CD44 in EC are still debated [31, 32]. In a previous study [10], our team found that patients with epithelial ovarian cancer with high ALDH1 expression were associated with CD44 expression, drug resistance, and poor clinical outcome. In the present study, we were eager to determine whether a higher expression of both ALDH1 and CD44 was associated with EC and clinical properties. To our surprise, we found a similar outcome revealed in EC. High levels of ALDH1 expression were found in 44.25% of our EC samples, which was significantly higher than in samples of pre-malignant lesions. A similar result was noted for CD44, with 35.40% of EC patients expressing this antigen, which was significantly higher than in cases of pre-malignant lesions. However, the majority of EC samples had low ALDH1 expression and were negative for CD44. These results suggest that the ALDH1^High^ and CD44^+^ cancer cells constitute only a relatively small fraction of cancer stem cells in EC.

CD44 can be found in various endometrial compartments of epithelial cells, and it plays important roles in the interactions between cancerous tissue and normal epithelium [33]. However, the actual function of CD44 in human cells is currently not fully understood.

There are some limitations of our research. These are primarily the small sample size (relative to the number of subgroups) and the inability to extrapolate the results to other populations, i.e., to different subgroups of patients that might have differences in other risk factors. Assessments using a combination of markers may provide better performance than when considering them individually. Therefore, we emphasize the prognostic value of the combined status of ALDH1^High^/CD44^+^ in EC. Our data from the combined analysis showed that 18.58% of EC cases displayed the ALDH1^High^/CD44^+^ phenotype, whereas 16.81% of cases had the ALDH1^Low^/CD44^+^ phenotype, and 25.66% expressed the ALDH1^High^/ CD44^-^ phenotype. These findings suggest that the ALDH1^High^ population and CD44^+^ EC cells may belong to two different subsets. Furthermore, we found that patients with EC cells presenting ALDH1^High^ or CD44^+^ had poor OS. However, the OS of patients with both CD44^+^ and ALDH1^High^ expression was even lower, which suggests that this combination can predict poor outcomes in EC.

In summary, the combined expression of ALDH1 and CD44 has been shown to identify subtypes of endometrial cancer with poor prognosis. Therefore, stratification of patients by the combined ALDH1^High^/CD44^+^ phenotype could be used to classify subgroups of EC patients for whom a more aggressive add-on therapy is mandatory.

Furthermore, using this combined expression, clinically beneficial agents for targeted delivery of diagnostic or therapeutic agents are promising and will likely be developed in the near future.

## Supporting information

S1 Table Correlation of ALDH1 in endometrial stroma cells between pre-malignant endometrial lesions and endometrial carcinoma. (DOCX)
