# Supplementary material for: The combination of aldehyde dehydrogenase 1 (ALDH1) and CD44 is associated with poor outcomes in endometrial cancer
Source: PLoS One. 2018 Oct 29;13(10):e0206685. doi: 10.1371/journal.pone.0206685 (PMC6205661; doi:10.1371/journal.pone.0206685)
Supplement: S1 Table — (DOCX) [file pone.0206685.s001.docx]

**S1 Table.**

| group | n | M±SE | P value | Bonferroni post hoc |
| --- | --- | --- | --- | --- |
| Normal | 42 | 68.21±13.46 |  | N vs. EC p<0.001  EH vs. N p<0.001,  AH vs. EC p=0.001  EH vs. EC p<0.001 ,EH vs. AH p<0.001 |
| EH | 48 | 121.33±12.98 |  |  |
| AH | 42 | 59.74±10.09 |  |  |
| EC | 113 | 17.50±2.45 | <0.001 |  |

M±SE=Mean±Standard Error

Normal: normal endometrium; EH: endometrial hyperplasia without atypia; AH: atypical hyperplasia; EC: endometrial carcinoma
